# Supplementary material for: Continued high rates of antibiotic prescribing to adults with respiratory tract infection: survey of 568 UK general practices
Source: BMJ Open. 2014 Oct 27;4(10):e006245. doi: 10.1136/bmjopen-2014-006245 (PMC4212213; doi:10.1136/bmjopen-2014-006245)
Supplement: Author's manuscript [file bmjopen-2014-006245.draft_revisions.pdf]

BMJ Open

Antibiotic prescribing to adults with respiratory tract infection at UK general practices: an update

Journal:	*BMJ Open*
Manuscript ID:	bmjopen-2014-006245
Article Type:	Research
Date Submitted by the Author:	01-Aug-2014

Complete List of Authors:	Gulliford, Martin; King's College London, UK, Primary Care and Public Health Sciences<br>Dregan, Alexandru; King's College London, UK, Primary Care and Public Health Sciences<br>Moore, Michael; University of Southampton Medical School, Primary Care Medical Group<br>Asthworth, Mark; King's College London, UK, Primary Care and Public Health Sciences<br>van Staa, Tjeerd; Medicines and Healthcare products Regulatory Agency, Clinical Practice Research Datalink (CPRD) Division<br>McCann, Gerard; Medicines and Healthcare products Regulatory Agency, Clinical Practice Research Datalink (CPRD) Division<br>Charlton, Judith; King's College London, UK, Primary Care and Public Health Sciences<br>Yardley, Lucy; University of Southampton, Academic Unit of Psychology<br>Little, Paul; University of Southampton, Primary Care and Population Science;<br>McDermott, Lisa; King's College London, Primary Care and Public Health Sciences

**Primary Subject Heading**:	Public health
Secondary Subject Heading:	Health services research
Keywords:	PUBLIC HEALTH, Health policy < HEALTH SERVICES ADMINISTRATION & MANAGEMENT, Clinical audit < HEALTH SERVICES ADMINISTRATION & MANAGEMENT

SCHOLARONE™
Manuscripts

Antibiotic prescribing to adults with respiratory tract infection at UK general practices: an update

Martin C. Gulliford[a] , Alex Dregan[a], Michael V. Moore[b], Mark Ashworth[a], Tjeerd van Staa[c,d], Gerard McCann[c], Judith Charlton[a], Lucy Yardley[b], Paul Little[b], Lisa McDermott[a]

[a] King's College London, Primary Care and Public Health Sciences, London, UK

[b] Primary Care and Population Sciences, University of Southampton, Southampton, UK

[c] Clinical Practice Research Datalink (CPRD) Division, Medicines and Healthcare products Regulatory Agency, London, UK;

[d]London School of Hygiene & Tropical Medicine, London, United Kingdom

Correspondence: Dr Lisa McDermott
Department of Primary Care and Public Health Sciences
King's College London
Capital House, 42 Weston St,
London SE1 3QD UK
Tel: 0207 848 6120
Fax: 0207 848 6620
E mail: lisa.mcdermott@kcl.ac.uk

Word count: Abstract: 250 words

Text: 1,499 words

Tables: 1

Figures: 1

For peer review only - http://bmjopen.bmj.com/site/about/guidelines.xhtml

Objectives: Over-utilisation of antibiotics may contribute to the emergence of antimicrobial drug resistance, a phenomenon of growing international concern. This study aimed to evaluate the performance of UK general practices with respect to antibiotic prescribing for respiratory tract infections among young and middle-aged adults.

Setting: Data are reported for 568 UK general practices contributing to the Clinical Practice Research Datalink.

Participants: Participants were adults aged 18 to 59 years. Consultations were identified for acute upper respiratory tract infections including colds, cough, otitis-media, rhino-sinusitis and sore throat.

Primary and secondary outcome measures: For each consultation, we identified whether an antibiotic was prescribed. The proportion of RTI consultations with antibiotics prescribed was estimated.

Results: There were 568 general practices analysed. The median general practice prescribed antibiotics at 54% of RTI consultations. At the highest prescribing 10% of practices, antibiotics were prescribed at 69% of RTI consultations. At the lowest prescribing 10% of practices, antibiotics were prescribed at 39% RTI consultations. The median practice prescribed antibiotics at 38% of consultations for ‘colds and upper respiratory tract infections’, 48% for ‘cough and bronchitis’, 60% for ‘sore throat’, 60% for ‘otitis-media’ and 91% for ‘rhino-sinusitis’. The highest prescribing 10% of practices issued antibiotic prescriptions at 72% of consultations for ‘colds’, 67% for ‘cough’, 78% for ‘sore throat’, 90% for ‘otitis-media’ and 100% for ‘rhino-sinusitis’.

Conclusions: Most UK general practices prescribe antibiotics to young and middle aged adults with respiratory infections at rates that are considerably in excess of what is clinically justified. This will fuel antibiotic resistance.

Key words: respiratory infections, antimicrobial drugs, primary care, implementation science

Strengths and limitations of this study

Strengths:

- The findings are derived from a large, representative sample of UK general practices.

For peer review only - http://bmjopen.bmj.com/site/about/guidelines.xhtml

Limitations:

- Findings did not include information concerning severity of illness or the presence of comorbidity, which might have accounted for the prescription of antibiotics in some cases.
- Only prescriptions issued by the practice were analysed, as it was not possible to estimate from electronic health records whether prescriptions were dispensed, or whether a delayed prescribing strategy was intended.

For peer review only

For peer review only - http://bmjopen.bmj.com/site/about/guidelines.xhtml

INTRODUCTION

Over use of antibiotic drugs is leading to increasing antimicrobial drug resistance. As there are now fewer new antibiotic drugs being developed, it is important to preserve the effectiveness of presently available antibiotics for future generations.[1] The Chief Medical Officer's annual report for 2011[1] promoted the concept of antimicrobial stewardship, which means that unnecessary or inappropriate use of antibiotics should be avoided so as to minimise the selection of antibiotic resistant strains of organisms.

In primary care, respiratory tract infections (RTI) are a common reason for consultation and antibiotics are frequently prescribed. RTIs account for about 60% of antibiotic prescribing in primary care.[2] Previous studies showed that antibiotic utilisation at consultations for respiratory infections declined during the 1990s but has remained constant since.[3] However, there has been a long term decline in the rate of consultation for RTI in UK primary care.[4]

In 2008, the National Institute for Health and Care Excellence (NICE) recommended that most acute respiratory tract infections, including colds, coughs, sore throats, otitis-media and rhinosinusitis, could be managed without antibiotics and recommended that either a 'no antibiotic' or 'delayed antibiotic prescribing' strategy should be agreed for most patients.[2]

We recently completed a large cluster randomised trial to reduce antibiotic prescribing among general practices that contribute to the Clinical Practice Research Datalink (CPRD).[5,6] In the present analysis, we aimed to describe the performance of UK general practices with respect to antibiotic prescribing for respiratory illness in young and middle-aged adults.

For peer review only - http://bmjopen.bmj.com/site/about/guidelines.xhtml

METHODS

The analysis included UK general practices that contributed to the UK Clinical Practice Research Datalink (CPRD). The study obtained Ethical approval from the South West London Research and Ethics Committee (09/H0806/81) and was approved by the CPRD Independent Scientific Advisory Committee (ISAC protocol 08_083). The period of study included the 12 months preceding the start date of the cluster trial. For the 104 practices included in the cluster trial, the date of random allocation was used as the index date. The practices were allocated in five batches between 26th November 2010 and 26th April 2011. For non-trial practices, the median of the allocation dates, 20th January 2011, was used as the index date. General practices that contributed fewer than 10 consultations for respiratory tract infections during the 12 month period were excluded.

Individual participants were adults aged 18 to 59 years. This was consistent with the eligibility criteria for the trial,[5] which aimed to exclude children and older adults who might be at higher risk of complications. All patients were included for the 104 practices that participated in the trial and, in order to provide a manageable dataset for analysis, a random sample of patients was taken from practices that did not participate in the trial. For each participant, we analysed their clinical record for 12 months before the trial index date date. In total there were 1,018,708 person years analysed, including 523,107 for trial practices and 495,601 person years for non-trial practices. The analysis used 232 Read codes, including those for ‘colds’ and ‘upper respiratory tract infection’ (URTI); ‘cough’ and ‘bronchitis’; ‘sore throat’, including pharyngitis, laryngitis, tracheitis, epiglottitis and tonsillitis; ‘otitis-media’ including acute otitis media and otitis media; and ‘rhino-sinusitis’ including all forms of sinusitis. These were used to identify consultations for acute respiratory tract infections. The source of information for RTI consultations was represented by clinical, referral, and test files data. Only first consultations within an episode were included using a 10 day time window. Therapy file data were used to ascertain antibiotic prescribing information. Antibiotic prescriptions were identified using drug codes that map to section 5.1 of the British National

For peer review only - http://bmjopen.bmj.com/site/about/guidelines.xhtml

Formulary, excluding drugs used to tuberculosis and leprosy. For each general practice, we estimated the consultation rate for RTI per 1,000 registered patients, the antibiotic prescribing rate per 1,000 registered patients, and the proportion of RTI consultations with antibiotics prescribed as reported previously.[4]

RESULTS

There were 582 CPRD general practices available for analysis, 14 practices which contributed fewer than 10 RTI consultations during the study period were excluded leaving 568 for further analysis. There were 431 practices in England, 21 in Northern Ireland, 66 in Scotland and 50 in Wales. Data were analysed for registered patients aged 18 to 59 years. There were 219,162 consultations for RTI by eligible patients available for analysis. There was a mean rate of 217 RTI consultations per 1,000 person years and a mean rate of 119 antibiotic prescriptions for RTI per 1,000 person years. Coefficients of variation of the practice-specific rates were 0.30 for the RTI consultation rate and 0.41 for the antibiotic prescribing rate respectively.

Figure 1 shows the distribution of the practice-specific proportion of RTI consultations with antibiotics prescribed for 568 UK general practices. Considering all RTI consultations as a single group, most practices prescribed antibiotics at between 30% and 80% of RTI consultations. There were only 18 (3%) of practices that prescribed antibiotics at fewer than 30% of RTI consultations and four (1%) of practices that prescribed antibiotics at fewer than 20% of RTI consultations.

Table 1 presents selected centiles of the distribution of the practice-specific prescribing proportions. The figures represent the percent of RTI consultations with antibiotics prescribed for the general practice that occupies the median (or quartile, decile etc) position

For peer review only - http://bmjopen.bmj.com/site/about/guidelines.xhtml

in the distribution of results for all 568 practices The median practice prescribed antibiotics at 54% of RTI consultations. The highest prescribing 10% of practices issued prescriptions at 69% or more of RTI consultations, and the highest prescribing 5% of practices issued prescriptions at 74% or more of all RTI consultations. By contrast, the lowest prescribing 10% of practices issued prescriptions at 39% of RTI consultations, and the lowest prescribing 5% of practices issued antibiotic prescriptions at 33% of RTI consultations.

Consultations for ‘cough and bronchitits’ accounted for 39% of RTI consultations; ‘sore throat’, 27%; ‘colds and URTI’, 19%; ‘rhino-sinusitis’ 9%; and ‘otitis media’ 6%. Table 2 shows the distribution of practice specific prescribing proportions according to the type of RTI consultation. The median practice issued antibiotic prescriptions at 38% of consultations for ‘colds’, 48% for ‘cough’, 60% for ‘otitis media’ and ‘sore throat’, and 91% for ‘rhino-sinusitis’. However, the highest prescribing 10% of practices issued antibiotic prescriptions at 72% of consultations for ‘colds and URTI’, 67% for ‘cough and bronchitis’, 78% for ‘sore throat’, 90% for ‘otitis-media’ and 100% for ‘rhino-sinusitis’. The lowest prescribing 10% of practices issued antibiotic prescriptions at 14% of consultations for ‘colds and URTI’, 28% for ‘cough’ and 41% for ‘sore throat’.

DISCUSSION

National guidance in the UK recommends that most patients presenting with acute respiratory tract infections can be managed with either no antibiotic prescribing or delayed antibiotic prescribing, with a prescription only being used if symptoms do not improve.[2] The present results show that most general practices in the UK depart substantially from recommended standards of good practice with respect to antibiotic prescribing in a generally low risk age range of young and middle-aged adults. Even for common colds and upper respiratory tract infections, which are generally acknowledged to have a viral aetiology,

For peer review only - http://bmjopen.bmj.com/site/about/guidelines.xhtml
For peer review only

antibiotics may be prescribed for a third of patients overall and for more than 80% of patients at some general practices. A number of trials have now shown that antibiotic prescribing may be reduced through educational interventions, together with feedback of prescribing information.[7-9] However, these interventions generally have modest effects with generally less than 10-15% reduction in antibiotic prescribing. As Linder[10] has observed, current antibiotic prescribing appears, to be ‘way off the mark’ when viewed in the context of systematic review evidence of lack of benefit[11] and current recommendations for good clinical practice.[2]

Our study had the strengths of a large, representative sample of UK general practices. We acknowledge that we did not include information concerning severity of illness or the presence of comorbidity, which might have accounted for the prescription of antibiotics in some cases. We only analysed prescriptions issued by the practice and it was not possible to estimate from electronic health records whether the prescription was dispensed, or whether a delayed prescribing strategy was intended. There is a Read code for deferred antibiotic therapy (8BP0.00) but this was recorded for fewer than 0.5% of medical events. It is unlikely that delayed prescribing can fully account for the high prescribing rates. In an observational study in 13,000 adults with sore throat immediate antibiotics were issued in 42% and 12% given delayed antibiotics.[12] Delayed prescribing is unlikely to vitiate our conclusion that most UK practices prescribe antibiotics to excess. One driver of prescribing is worries about complications but complications are hard to predict and rare and that delayed prescribing is probably as effective as immediate prescribing to reduce the risk of complications.[12]

The present results have implications for communications with the public as well as for practice prescribing policies. Respiratory infections in this age group are both self-limiting and at low risk of complications, moreover the impact of antibiotics on symptom severity and duration is at best marginal. Respiratory infections may be better managed through patient self-care rather than primary care consultation. The high rates of antibiotic prescribing

For peer review only

reported by this study indicate a need to shift the entire distribution for antibiotic prescribing to lower levels, since there are very few practices that are not prescribing antibiotics to excess, fuelling the development of antibiotic resistance. There needs to be an active professional debate concerning an overall level of antibiotic utilisation for RTI that might be acceptable, and the size of reduction that individual practices should aim to achieve as a matter of urgency.

Funding:

The study was supported by the Joint Initiative in Electronic Patient Records and Databases in Research, a partnership between the Wellcome Trust, Medical Research Council, Economics & Social Research Council and Engineering & Physical Sciences Research Council (WT086129MA). MG and AD were supported by the National Institute for Health Research (NIHR) Biomedical Research Centre at Guy's and St Thomas' NHS Foundation Trust and King's College London. The views expressed are those of the author(s) and not necessarily those of the NHS, the NIHR or the Department of Health. CPRD has received funding from the MHRA, Wellcome Trust, Medical Research Council, NIHR Health Technology Assessment programme, Innovative Medicine Initiative, UK Department of Health, Technology Strategy Board, Seventh Framework Programme EU, various universities, contract research organisations and pharmaceutical companies. The views expressed in this paper are those of the authors and do not reflect the official policy or position of the MHRA.

Competing interests:

The authors declare that they have no competing interests.

For peer review only

For peer review only - http://bmjopen.bmj.com/site/about/guidelines.xhtml

Contributor statement:

MG designed, supervised, and drafted the paper. LM assisted with draft and conclusions. JC and AD contributed to data analysis. lysis. MA, MM, PL, LY contributed to design, write-up and interpretation of data. TvS and GM contributed to practice recruitment and facilitated access to CPRD. All authors contributed to the paper and approved the final draft. All authors read and approved the final manuscript.

Data sharing:

There is no data sharing available at present.

For peer review only

REFERENCES

1. Chief Medical Officer. *Annual report of the cheif medical officer. Volume 2, 2011. Infections and the rise of antimicrobial resistance*. London: Department of Health, 2013

2. National Institute for Health and Clinical Excellence. *Prescribing of antibiotics for self-limiting respiratory tract infections in adults and children in primary care.* London: National Institute for Health and Clinical Excellence; 2008.

3. Ashworth M, Latinovic R, Charlton J, Cox K, Rowlands G, Gulliford M. Why has antibiotic prescribing for respiratory illness declined in primary care? A longitudinal study using the general practice research database. *J Public Health (Oxf).* 2004;**26:**268-274

4. Gulliford M, Latinovic R, Charlton J, Little P, van Staa T, Ashworth M. Selective decrease in consultations and antibiotic prescribing for acute respiratory tract infections in uk primary care up to 2006. *Journal of Public Health*. 2009;**31**:512-520

5. Gulliford MC, van Staa T, McDermott L, Dregan A, McCann G, Ashworth M, Charlton J, Grieve AP, Little P, Moore MV, Yardley L. Cluster randomised trial in the general practice research database: 1. Electronic decision support to reduce antibiotic prescribing in primary care (eCRT study). *Trials*. 2011;**12**:115

6. Gulliford M, van Staa T, Dregan A, McDermott L, McCann G, Ashworth M, Charlton J, Little P, Moore MV, Yardley L. Utilising electronic health records for intervention research. Cluster randomised trial to reduce antibiotic prescribing in primary care (eCRT study). *Annals of Family Medicine*. 2014;In Press

7. Little P, Stuart B, Francis N, Douglas E, Tonkin-Crine S, Anthierens S, Cals JW, Melbye H, Santer M, Moore M, Coenen S, Butler C, Hood K, Kelly M, Godycki-Cwirko M, Mierzecki A, Torres A, Llor C, Davies M, Mullee M, O'Reilly G, van der Velden A, Geraghty AW, Goossens H, Verheij T, Yardley L. Effects of internet-based training on antibiotic prescribing rates for acute respiratory-tract infections: A

For peer review only - http://bmjopen.bmj.com/site/about/guidelines.xhtml

multinational, cluster, randomised, factorial, controlled trial. *Lancet*. 2013;**382**:1175-1182

8. Butler CC, Simpson SA, Dunstan F, Rollnick S, Cohen D, Gillespie D, Evans MR, Alam MF, Bekkers MJ, Evans J, Moore L, Howe R, Hayes J, Hare M, Hood K. Effectiveness of multifaceted educational programme to reduce antibiotic dispensing in primary care: Practice based randomised controlled trial. *BMJ*. 2012;**344**:d8173

9. Gonzales R, Anderer T, McCulloch CE, Maselli JH, Bloom FJ Jr, Graf TR, Stahl M, Yefko M, Molecavage J, Metlay JP. A cluster randomized trial of decision support strategies for reducing antibiotic use in acute bronchitis. *JAMA Internal Medicine*. 2013;**173**:267-273

10. Linder JA. Antibiotic prescribing for acute respiratory infections--success that's way off the mark: Comment on "a cluster randomized trial of decision support strategies for reducing antibiotic use in acute bronchitis". *JAMA Intern Med*. 2013;**173**:273-275

11. Del Mar CB, Glasziou PP, Spinks AB. Antibiotics for sore throat. *Cochrane.Database.Syst.Rev.* 2000:CD000023

12. Little P, Stuart B, Hobbs FD, Butler CC, Hay AD, Delaney B, Campbell J, Broomfield S, Barratt P, Hood K, Everitt H, Mullee M, Williamson I, Mant D, Moore M. Antibiotic prescription strategies for acute sore throat: A prospective observational cohort study. *Lancet Infect Dis*. 2014;**14**:213-219

For peer review only

For peer review only - http://bmjopen.bmj.com/site/about/guidelines.xhtml

Table 1: Centiles of the distribution of the proportion (%) of RTI consultations with antibiotics prescribed at 568 UK general practices.

	Proportion of RTI consultations with antibiotics prescribed at UK general practices						
	Lowest 5% of practices	Lowest 10%	Lowest 25%	Median	Highest 25%	Highest 10%	Highest 5% of practices
All	33	39	46	54	63	69	74
'Colds' and 'URTI'	9	14	25	38	56	72	81
'Cough and bronchitis'	22	28	38	48	59	67	71
'Otitis-media'	22	32	45	60	75	90	100
'Rhino-sinusitis'	67	75	83	91	98	100	100
'Sore throat'	35	41	50	60	68	78	83

URTI, upper respiratory tract infection
Note that one practice may not occupy the same centile of prescribing for each condition.

For peer review only

For peer review only - http://bmjopen.bmj.com/site/about/guidelines.xhtml

Figure 1: Distribution for percent of RTI consultations with antibiotics prescribed for adults aged 18 to 59 years at 568 UK general practices.

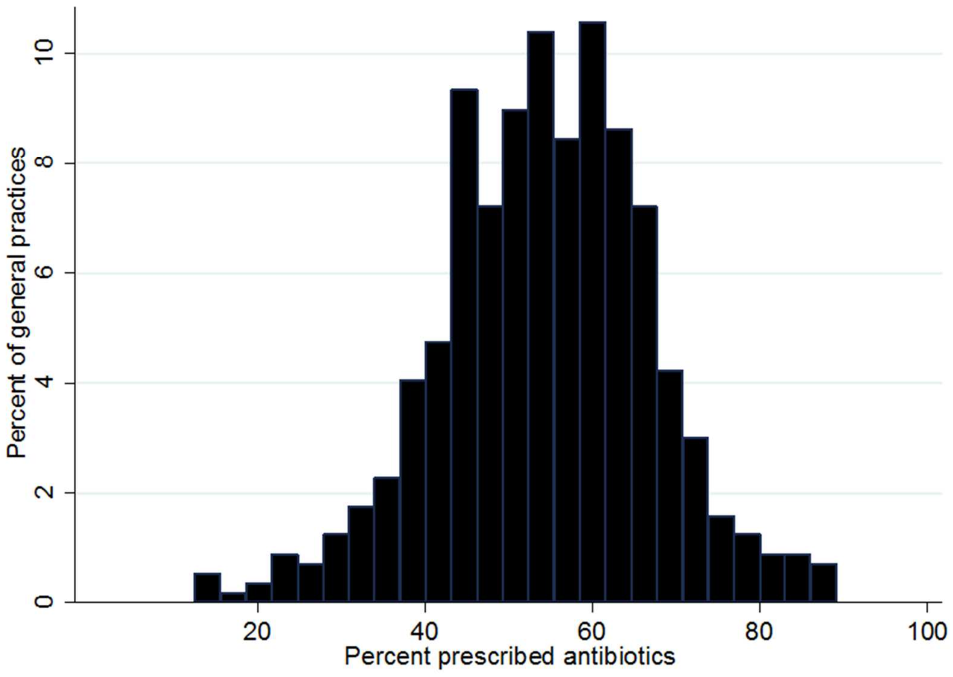

For peer review only - http://bmjopen.bmj.com/site/about/guidelines.xhtml

Continued high rates of antibiotic prescribing to adults with respiratory tract infection. Survey of 568 UK general practices

Journal:	*BMJ Open*
Manuscript ID:	bmjopen-2014-006245.R1
Article Type:	Research
Date Submitted by the Author:	18-Sep-2014

Complete List of Authors:	Gulliford, Martin; King's College London, UK, Primary Care and Public Health Sciences<br>Dregan, Alexandru; King's College London, UK, Primary Care and Public Health Sciences<br>Moore, Michael; University of Southampton Medical School, Primary Care Medical Group<br>Asthworth, Mark; King's College London, UK, Primary Care and Public Health Sciences<br>van Staa, Tjeerd; Medicines and Healthcare products Regulatory Agency, Clinical Practice Research Datalink (CPRD) Division<br>McCann, Gerard; Medicines and Healthcare products Regulatory Agency, Clinical Practice Research Datalink (CPRD) Division<br>Charlton, Judith; King's College London, UK, Primary Care and Public Health Sciences<br>Yardley, Lucy; University of Southampton, Academic Unit of Psychology<br>Little, Paul; University of Southampton, Primary Care and Population Science;<br>McDermott, Lisa; King's College London, Primary Care and Public Health Sciences

**Primary Subject Heading**:	Public health
Secondary Subject Heading:	Health services research
Keywords:	PUBLIC HEALTH, Health policy < HEALTH SERVICES ADMINISTRATION & MANAGEMENT, Clinical audit < HEALTH SERVICES ADMINISTRATION & MANAGEMENT

Continued high rates of antibiotic prescribing to adults with respiratory tract infection. Survey of 568 UK general practices

Martin C. Gulliford[a] , Alex Dregan[a], Michael V. Moore[b], Mark Ashworth[a], Tjeerd van Staa[c,d], Gerard McCann[c], Judith Charlton[a], Lucy Yardley[b], Paul Little[b], Lisa McDermott[a]

[a] King's College London, Primary Care and Public Health Sciences, London, UK

[b] Primary Care and Population Sciences, University of Southampton, Southampton, UK

[c] Clinical Practice Research Datalink (CPRD) Division, Medicines and Healthcare products Regulatory Agency, London, UK;

[d] Health eResearch Centre, Farr Institute for Health Informatics Research, University of Manchester

Correspondence: Dr Lisa McDermott
Department of Primary Care and Public Health Sciences
King's College London
Capital House, 42 Weston St,
London SE1 3QD UK
Tel: 0207 848 6120
Fax: 0207 848 6620
E mail: lisa.mcdermott@kcl.ac.uk

Word count: Abstract: 250 words

Text: 1,893 words

Tables: 1

Figures: 2

For peer review only

For peer review only - http://bmjopen.bmj.com/site/about/guidelines.xhtml

Objectives: Over-utilisation of antibiotics may contribute to the emergence of antimicrobial drug resistance, a growing international concern. This study aimed to analyse the performance of UK general practices with respect to antibiotic prescribing for respiratory tract infections among young and middle-aged adults.

Setting: Data are reported for 568 UK general practices contributing to the Clinical Practice Research Datalink.

Participants: Participants were adults aged 18 to 59 years. Consultations were identified for acute upper respiratory tract infections including colds, cough, otitis-media, rhino-sinusitis and sore throat.

Primary and secondary outcome measures: For each consultation, we identified whether an antibiotic was prescribed. The proportion of RTI consultations with antibiotics prescribed was estimated.

Results: There were 568 general practices analysed. The median general practice prescribed antibiotics at 54% of RTI consultations. At the highest prescribing 10% of practices, antibiotics were prescribed at 69% of RTI consultations. At the lowest prescribing 10% of practices, antibiotics were prescribed at 39% RTI consultations. The median practice prescribed antibiotics at 38% of consultations for 'colds and upper respiratory tract infections', 48% for 'cough and bronchitis', 60% for 'sore throat', 60% for 'otitis-media' and 91% for 'rhino-sinusitis'. The highest prescribing 10% of practices issued antibiotic prescriptions at 72% of consultations for 'colds', 67% for 'cough', 78% for 'sore throat', 90% for 'otitis-media' and 100% for 'rhino-sinusitis'.

Conclusions: Most UK general practices prescribe antibiotics to young and middle aged adults with respiratory infections at rates that are considerably in excess of what is clinically justified. This will fuel antibiotic resistance.

Key words: respiratory infections, antimicrobial drugs, primary care, implementation science

For peer review only - http://bmjopen.bmj.com/site/about/guidelines.xhtml

Strengths and limitations of this study

Strengths:

- The findings are derived from a large, representative sample of UK general practices.

Limitations:

- Findings did not include information concerning severity of illness or the presence of comorbidity, which might have accounted for the prescription of antibiotics in some cases.
- Only prescriptions issued by the practice were analysed, as it was not possible to estimate from electronic health records whether prescriptions were dispensed, or whether a delayed prescribing strategy was intended.

For peer review only

For peer review only - http://bmjopen.bmj.com/site/about/guidelines.xhtml

INTRODUCTION

Over use of antibiotic drugs is leading to increasing antimicrobial drug resistance. As there are now fewer new antibiotic drugs being developed, it is important to preserve the effectiveness of presently available antibiotics for future generations.[1] The Chief Medical Officer's annual report for 2011[1] promoted the concept of antimicrobial stewardship, which means that unnecessary or inappropriate use of antibiotics should be avoided so as to minimise the selection of antibiotic resistant strains of organisms. In addition to increasing antimicrobial drug resistance, the over use of antibiotic drugs can lead to unnecessary side-effects and increase future consultations for RTI's.[2 3] In primary care, respiratory tract infections (RTI) are a common reason for consultation and antibiotics are frequently prescribed. RTIs account for about 60% of antibiotic prescribing in primary care.[4] Previous studies showed that antibiotic utilisation at consultations for respiratory infections declined during the 1990s but has remained constant since.[5] However, there has been a long term decline in the rate of consultation for RTI in UK primary care.[6]

In 2008, the National Institute for Health and Care Excellence (NICE) recommended that most acute respiratory tract infections, including colds, coughs, sore throats, otitis-media and rhinosinusitis, could be managed without antibiotics and recommended that either a 'no antibiotic' or 'delayed antibiotic prescribing' strategy should be agreed for most patients.[4]

We recently completed a large cluster randomised trial to reduce antibiotic prescribing among general practices that contribute to the Clinical Practice Research Datalink (CPRD).[7, 8] The study aimed to reduce unnecessary antibiotic prescribing using an electronically delivered intervention. The present analysis included data from general practices that participated in the trial as well as data from non-trial general practices. We aimed to describe

For peer review only - http://bmjopen.bmj.com/site/about/guidelines.xhtml

the performance of UK general practices with respect to antibiotic prescribing for respiratory illness in young and middle-aged adults.

METHODS

The UK Clinical Practice Research Datalink (CPRD) provided the data source for the study. The CPRD is a database of prospectively collected electronic medical records from approximately 7% of UK general practices. It includes records for all prescriptions issued and medical diagnoses recorded.[9] The study obtained Ethical approval from the South West London Research and Ethics Committee (09/H0806/81) and was approved by the CPRD Independent Scientific Advisory Committee (ISAC protocol 08_083). The study included all CPRD general practices that were included in the cluster trial,[8] as well as sample data for all CPRD general practices that were not included in the trial. All registered patients were included for the trial practices and, in order to provide a manageable dataset for analysis, a random sample of registered patients was taken from non-trial practices. The period of study included the 12 months preceding the start date of the cluster trial withthe date of random allocation was used as the index date.[8] The practices were allocated in five batches between 26th November 2010 and 26th April 2011. For non-trial practices, the median of the allocation dates, 20th January 2011, was used as the index date.

Individual participants were adults aged 18 to 59 years. This was consistent with the eligibility criteria for the trial,[7] which aimed to exclude children and older adults who might be at higher risk of complications. For each participant, we analysed their clinical record for 12 months before the trial index date. General practices were analysed as a single group as there were no overall baseline differences between trial and non-trial practices with respect to consultation and antibiotic prescribing rates.[8] The analysis used 232 general practice Read medical codes (recorded by GPs for each patient who consulted with a RTI), including those for ‘colds’ and ‘upper respiratory tract infection’ (URTI); ‘cough’ and ‘bronchitis’; ‘sore

For peer review only - http://bmjopen.bmj.com/site/about/guidelines.xhtml

throat', including pharyngitis, laryngitis, tracheitis, epiglottitis and tonsillitis; 'otitis-media' including acute otitis media and otitis media; and 'rhino-sinusitis' including all forms of sinusitis. These were used to identify consultations for acute respiratory tract infections. The source of information for RTI consultations was represented by clinical, referral, and test files data. Only first consultations within an episode were included using a 10 day time window. Therapy file data were used to ascertain antibiotic prescribing information. Antibiotic prescriptions were identified using drug codes that map to section 5.1 of the British National Formulary, excluding drugs used to tuberculosis and leprosy. For each general practice, we estimated the consultation rate for RTI per 1,000 registered patients, the antibiotic prescribing rate per 1,000 registered patients, and the proportion of RTI consultations with antibiotics prescribed as reported previously.[6]

RESULTS

The selection of general practices and patients into the analysis is outlined in Figure 1. There were 582 CPRD general practices available for analysis, 14 practices which contributed fewer than 10 RTI consultations during the study period were excluded leaving 568 for further analysis, including 101 that participated in the trial and 467 that did not participate in the trial. There were 431 practices in England, 21 in Northern Ireland, 66 in Scotland and 50 in Wales. Data were analysed for registered patients aged 18 to 59 years. There were 1,016,779 registered patients with 219,162 consultations for RTI and 118,583 antibiotic prescriptions available for analysis. There was a mean rate of 217 RTI consultations per 1,000 person years and a mean rate of 119 antibiotic prescriptions for RTI per 1,000 person years. Coefficients of variation of the practice-specific rates were 0.30 for the RTI consultation rate and 0.41 for the antibiotic prescribing rate respectively.

For peer review only - http://bmjopen.bmj.com/site/about/guidelines.xhtml

For peer review only

Figure 2 shows the distribution of the practice-specific proportion of RTI consultations with antibiotics prescribed for 568 UK general practices. Considering all RTI consultations as a single group, most practices prescribed antibiotics at between 30% and 80% of RTI consultations. There were only 18 (3%) of practices that prescribed antibiotics at fewer than 30% of RTI consultations and four (1%) of practices that prescribed antibiotics at fewer than 20% of RTI consultations.

Table 1 shows the distribution of the practice-specific prescribing proportions according to the type of RTI consultation. The figures represent the percent of RTI consultations with antibiotics prescribed for the general practice that occupies the stated position in the distribution of results for all 568 practices The median practice prescribed antibiotics at 54% of RTI consultations. The highest prescribing 10% of practices issued prescriptions at 69% or more of RTI consultations, and the highest prescribing 5% of practices issued prescriptions at 74% or more of all RTI consultations. By contrast, the lowest prescribing 10% of practices issued prescriptions at 39% of RTI consultations, and the lowest prescribing 5% of practices issued antibiotic prescriptions at 33% of RTI consultations.

Consultations for ‘cough and bronchitits’ accounted for 39% of RTI consultations; ‘sore throat’, 27%; ‘colds and URTI’, 19%; ‘rhino-sinusitis’ 9%; and ‘otitis media’ 6%. Table 1 shows the distribution of practice specific prescribing proportions according to the type of RTI consultation. The median practice issued antibiotic prescriptions at 38% of consultations for ‘colds’, 48% for ‘cough’, 60% for ‘otitis media’ and ‘sore throat’, and 91% for ‘rhino-sinusitis’. However, the highest prescribing 10% of practices issued antibiotic prescriptions at 72% of consultations for ‘colds and URTI’, 67% for ‘cough and bronchitis’, 78% for ‘sore throat’, 90% for ‘otitis-media’ and 100% for ‘rhino-sinusitis’. The lowest prescribing 10% of

For peer review only - http://bmjopen.bmj.com/site/about/guidelines.xhtml

For peer review only

practices issued antibiotic prescriptions at 14% of consultations for 'colds and URTI', 28% for 'cough' and 41% for 'sore throat'.

DISCUSSION

National guidance in the UK recommends that most patients presenting with acute respiratory tract infections can be managed with either no antibiotic prescribing or delayed antibiotic prescribing, with a prescription only being used if symptoms do not improve.[4] The present results show that most general practices in the UK depart substantially from recommended standards of good practice with respect to antibiotic prescribing in a generally low risk age range of young and middle-aged adults. Even for common colds and upper respiratory tract infections, which are generally acknowledged to have a viral aetiology, antibiotics may be prescribed for a third of patients overall and for more than 80% of patients at some general practices. A number of trials have now shown that antibiotic prescribing may be reduced through educational interventions, together with feedback of prescribing information.[10-12] However, these interventions generally have modest effects with generally less than 10-15% reduction in antibiotic prescribing. As Linder[13] has observed, current antibiotic prescribing appears, to be 'way off the mark' when viewed in the context of systematic review evidence of lack of benefit[14] and current recommendations for good clinical practice.[4]

Our study had the strengths of a large, representative sample of UK general practices. We acknowledge that we did not include information concerning severity of illness or the presence of comorbidity, which might have accounted for the prescription of antibiotics in some cases. We only analysed prescriptions issued by the practice and it was not possible to estimate from electronic health records whether the prescription was dispensed, or whether a delayed prescribing strategy was intended. There is a Read code for deferred

For peer review only - http://bmjopen.bmj.com/site/about/guidelines.xhtml

antibiotic therapy (8BP0.00) but this was recorded for fewer than 0.5% of medical events. It is unlikely that delayed prescribing can fully account for the high prescribing rates. In an observational study in 13,000 adults with sore throat immediate antibiotics were issued in 42% and 12% given delayed antibiotics.[15] Delayed prescribing is unlikely to vitiate our conclusion that most UK practices prescribe antibiotics to excess. One driver of prescribing is worries about complications but complications are hard to predict and rare and that delayed prescribing is probably as effective as immediate prescribing to reduce the risk of complications.[12] The categories used for analysis may have combined several different entities, for example, prescribing may be more frequent for cases coded as 'bronchitis' than for 'cough'. Prescribing for sinusitis was generally high, even at lower prescribing practices. We have not analysed practice characteristics as possible predictors of antibiotic prescribing, but such analyses typically only explain a small proportion of the variation between practices.[16] The results suggest that most practices commonly prescribe antibiotics unnecessarily. Patient characteristics such as age,[17] gender, comorbidity, smoking status or deprivation category might also be associated with prescribing decisions. Nevertheless, these results suggest that many patients may be prescribed antibiotics unnecessarily. Reducing antibiotic prescribing may lead to lower consultation rates for RTI.[18] The present study did not include children who represent some of the highest users of antibiotic prescriptions[17] but children will be included in a planned cluster randomised trial in CPRD to start in 2015.

The present results have implications for communications with the public as well as for practice prescribing policies. Respiratory infections in this age group are both self-limiting and carry a low risk of complications, moreover the impact of antibiotics on symptom severity and duration is at best marginal. Respiratory infections may be better managed through patient self-care rather than primary care consultation. The high rates of antibiotic prescribing reported by this study indicate a need to shift the entire distribution for antibiotic

For peer review only - http://bmjopen.bmj.com/site/about/guidelines.xhtml

prescribing to lower levels, since there are very few practices that are not prescribing antibiotics to excess, fuelling the development of antibiotic resistance. In addition, there are immediate direct costs from prescribing antibiotics, as well as risks of drug side effects and the perpetuation of unnecessary consultation patterns. There needs to be an active professional debate concerning an overall level of antibiotic utilisation for RTI that might be acceptable, and the size of reduction that individual practices should aim to achieve as a matter of urgency.

Funding:

The study was supported by the Joint Initiative in Electronic Patient Records and Databases in Research, a partnership between the Wellcome Trust, Medical Research Council, Economics & Social Research Council and Engineering & Physical Sciences Research Council (WT086129MA). MG and AD were supported by the National Institute for Health Research (NIHR) Biomedical Research Centre at Guy's and St Thomas' NHS Foundation Trust and King's College London. The views expressed are those of the author(s) and not necessarily those of the NHS, the NIHR or the Department of Health. CPRD has received funding from the MHRA, Wellcome Trust, Medical Research Council, NIHR Health Technology Assessment programme, Innovative Medicine Initiative, UK Department of Health, Technology Strategy Board, Seventh Framework Programme EU, various universities, contract research organisations and pharmaceutical companies. The views expressed in this paper are those of the authors and do not reflect the official policy or position of the MHRA.

For peer review only - http://bmjopen.bmj.com/site/about/guidelines.xhtml

Competing interests:

The authors declare that they have no competing interests.

Contributor statement:

MG designed, supervised, and drafted the paper. LM assisted with draft and conclusions. JC and AD contributed to data analysis. MA, MM, PL, LY contributed to design, write-up and interpretation of data. TvS and GM contributed to practice recruitment and facilitated access to CPRD. All authors contributed to the paper and approved the final draft. All authors read and approved the final manuscript.

Data sharing:

No additional data available

Figure 1. Flowchart showing selection of general practices and participants. (IQR, interquartile range).

Figure 2: Distribution for percent of RTI consultations with antibiotics prescribed for adults aged 18 to 59 years at 568 UK general practices.

For peer review only

REFERENCES

1. Chief Medical Officer. *Annual Report of the Chief Medical Officer. Volume 2, 2011. Infections and the rise of antimicrobial resistance.* London: Department of Health, 2013.

2. Little P, Gould C, Williamson I et al. Reattendance and complications in a randomised trial of prescribing strategies for sore throat: the medicalising effect of prescribing antibiotics. *BMJ*. 1997;**315**:350-352.

3. Glasziou PP, Del Mar CB, Sanders SL et al.. Antibiotics for acute otitis media in children. *The Cochrane database of systematic reviews*. 2004 (1):CD000219.

4. National Institute for Health and Care Excellence. *Prescribing of antibiotics for self-limiting respiratory tract infections in adults and children in primary care. Final scope 130907*. London: National Institute for Health and Care Excellence; 2008.

5. Ashworth M, Latinovic R, Charlton J et al. Why has antibiotic prescribing for respiratory illness declined in primary care? A longitudinal study using the General Practice Research Database. *J Public Health*. 2004;**26**:268-274.

6. Gulliford M, Latinovic R, Charlton J et al. Selective decrease in consultations and antibiotic prescribing for acute respiratory tract infections in UK primary care up to 2006. *J Public Health*. 2009;**31**:512-520.

7. Gulliford MC, van Staa T, McDermott L et al. Cluster randomised trial in the General Practice Research Database: 1. Electronic decision support to reduce antibiotic prescribing in primary care (eCRT study). *Trials*. 2011;12:115.

8. Gulliford M, van Staa T, Dregan A et al. Utilising electronic health records for intervention research. Cluster randomised trial to reduce antibiotic prescribing in primary care (eCRT study). *Annals of Family Medicine*. 2014;**12**: 344-51.

For peer review only - http://bmjopen.bmj.com/site/about/guidelines.xhtml

9. Williams T, van Staa T, Puri S et al.. Recent advances in the utility and use of the General Practice Research Database as an example of a UK Primary Care Data resource. *Therapeutic Advances in Drug Safety*. 2012;**3:**89-99.

10. Little P, Stuart B, Francis N et al. Effects of internet-based training on antibiotic prescribing rates for acute respiratory-tract infections: a multinational, cluster, randomised, factorial, controlled trial. *Lancet*. 2013;**382**:1175-82.

11. Butler CC, Simpson SA, Dunstan F et al. Effectiveness of multifaceted educational programme to reduce antibiotic dispensing in primary care: practice based randomised controlled trial. *BMJ*. 2012;**344**:d8173.

12. Gonzales R, Anderer T, McCuloch CE et al. A cluster randomized trial of decision support strategies for reducing antibiotic use in acute bronchitis. *JAMA Internal Medicine*. 2013;**173**:267-273.

13. Linder JA. Antibiotic Prescribing for acute respiratory infections--success that's way off the mark: comment on "A cluster randomized trial of decision support strategies for reducing antibiotic use in acute bronchitis". *JAMA Intern Med*. 2013;**173**:273-5.

14. Del Mar CB, Glasziou PP and Spinks AB. Antibiotics for sore throat. *Cochrane Database Syst Rev*. 2000:CD000023.

15. Little P, Stuart B, Hobbs FD et al. Antibiotic prescription strategies for acute sore throat: a prospective observational cohort study. *Lancet Infect Dis*. 2014;**14**:213-9.

16. Boggon R, Hubbard R, Smeeth L et al. Variability of antibiotic prescribing in patients with chronic obstructive pulmonary disease exacerbations: a cohort study. *BMC Pulmonary Medicine*. 2013;**13**:32.

17. Ashworth M, Charlton J, Latinovic R et al. Age-related changes in consultations and antibiotic prescribing for acute respiratory infections, 1995–2000. Data from the UK General

For peer review only - http://bmjopen.bmj.com/site/about/guidelines.xhtml

For peer review only

Practice Research Database. *Journal of Clinical Pharmacy and Therapeutics*. 2006;**31**:461-467.

18. Ashworth M, Charlton J, Ballard K et al. Variations in antibiotic prescribing and consultation rates for acute respiratory infection in UK general practices 1995-2000. *BrJ GenPract*. 2005;**55**:603-608.

For peer review only

For peer review only - http://bmjopen.bmj.com/site/about/guidelines.xhtml

Table 1: Centiles of the distribution of the proportion (%) of RTI consultations with antibiotics prescribed at 568 UK general practices.

	Proportion of RTI consultations with antibiotics prescribed at UK general practices						
	**Lowest 5% of practices**	**Lowest 10%**	**Lowest 25%**	**Median**	**Highest 25%**	**Highest 10%**	**Highest 5% of practices**
All	33	39	46	54	63	69	74
'Colds' and 'URTI'	9	14	25	38	56	72	81
'Cough and bronchitis'	22	28	38	48	59	67	71
'Otitis-media'	22	32	45	60	75	90	100
'Rhino-sinusitis'	67	75	83	91	98	100	100
'Sore throat'	35	41	50	60	68	78	83

URTI, upper respiratory tract infection
Note that one practice may not occupy the same centile of prescribing for each condition.

For peer review only

For peer review only - http://bmjopen.bmj.com/site/about/guidelines.xhtml

For peer review only

For peer review only - http://bmjopen.bmj.com/site/about/guidelines.xhtml

Continued high rates of antibiotic prescribing to adults with respiratory tract infection at UK general practices

Martin C. Gulliford[a] , Alex Dregan[a], Michael V. Moore[b], Mark Ashworth[a], Tjeerd van Staa[c,d], Gerard McCann[c], Judith Charlton[a], Lucy Yardley[b], Paul Little[b], Lisa McDermott[a]

[a] King's College London, Primary Care and Public Health Sciences, London, UK

[b] Primary Care and Population Sciences, University of Southampton, Southampton, UK

[c] Clinical Practice Research Datalink (CPRD) Division, Medicines and Healthcare products Regulatory Agency, London, UK;

[d] Health eResearch Centre, Farr Institute for Health Informatics Research, University of Manchester

Correspondence: Dr Lisa McDermott
Department of Primary Care and Public Health Sciences
King's College London
Capital House, 42 Weston St,
London SE1 3QD UK
Tel: 0207 848 6120
Fax: 0207 848 6620
E mail: lisa.mcdermott@kcl.ac.uk

Word count: Abstract: 250 words

Text: 1,893 words

Tables: 1

Figures: 2

For peer review only

Objectives: Over-utilisation of antibiotics may contribute to the emergence of antimicrobial drug resistance, a growing international concern. This study aimed to analyse the performance of UK general practices with respect to antibiotic prescribing for respiratory tract infections among young and middle-aged adults.

Setting: Data are reported for 568 UK general practices contributing to the Clinical Practice Research Datalink.

Participants: Participants were adults aged 18 to 59 years. Consultations were identified for acute upper respiratory tract infections including colds, cough, otitis-media, rhino-sinusitis and sore throat.

Primary and secondary outcome measures: For each consultation, we identified whether an antibiotic was prescribed. The proportion of RTI consultations with antibiotics prescribed was estimated.

Results: There were 568 general practices analysed. The median general practice prescribed antibiotics at 54% of RTI consultations. At the highest prescribing 10% of practices, antibiotics were prescribed at 69% of RTI consultations. At the lowest prescribing 10% of practices, antibiotics were prescribed at 39% RTI consultations. The median practice prescribed antibiotics at 38% of consultations for ‘colds and upper respiratory tract infections’, 48% for ‘cough and bronchitis’, 60% for ‘sore throat’, 60% for ‘otitis-media’ and 91% for ‘rhino-sinusitis’. The highest prescribing 10% of practices issued antibiotic prescriptions at 72% of consultations for ‘colds’, 67% for ‘cough’, 78% for ‘sore throat’, 90% for ‘otitis-media’ and 100% for ‘rhino-sinusitis’.

Conclusions: Most UK general practices prescribe antibiotics to young and middle aged adults with respiratory infections at rates that are considerably in excess of what is clinically justified. This will fuel antibiotic resistance.

Key words: respiratory infections, antimicrobial drugs, primary care, implementation science

For peer review only

Strengths and limitations of this study

Strengths:

- The findings are derived from a large, representative sample of UK general practices.

Limitations:

- Findings did not include information concerning severity of illness or the presence of comorbidity, which might have accounted for the prescription of antibiotics in some cases.
- Only prescriptions issued by the practice were analysed, as it was not possible to estimate from electronic health records whether prescriptions were dispensed, or whether a delayed prescribing strategy was intended.

For peer review only

For peer review only - http://bmjopen.bmj.com/site/about/guidelines.xhtml

INTRODUCTION

Over use of antibiotic drugs is leading to increasing antimicrobial drug resistance. As there are now fewer new antibiotic drugs being developed, it is important to preserve the effectiveness of presently available antibiotics for future generations.[1] The Chief Medical Officer's annual report for 2011[1] promoted the concept of antimicrobial stewardship, which means that unnecessary or inappropriate use of antibiotics should be avoided so as to minimise the selection of antibiotic resistant strains of organisms. In addition to increasing antimicrobial drug resistance, the over use of antibiotic drugs can lead to unnecessary side-effects and increase future consultations for RTI's.[2 3] In primary care, respiratory tract infections (RTI) are a common reason for consultation and antibiotics are frequently prescribed. RTIs account for about 60% of antibiotic prescribing in primary care.[4] Previous studies showed that antibiotic utilisation at consultations for respiratory infections declined during the 1990s but has remained constant since.[5] However, there has been a long term decline in the rate of consultation for RTI in UK primary care.[6]

In 2008, the National Institute for Health and Care Excellence (NICE) recommended that most acute respiratory tract infections, including colds, coughs, sore throats, otitis-media and rhinosinusitis, could be managed without antibiotics and recommended that either a 'no antibiotic' or 'delayed antibiotic prescribing' strategy should be agreed for most patients.[4]

We recently completed a large cluster randomised trial to reduce antibiotic prescribing among general practices that contribute to the Clinical Practice Research Datalink (CPRD).[7, 8] The study aimed to reduce unnecessary antibiotic prescribing using an electronically delivered intervention. The present analysis included data from general practices that participated in the trial as well as data from non-trial general practices. We aimed to describe

For peer review only - http://bmjopen.bmj.com/site/about/guidelines.xhtml

the performance of UK general practices with respect to antibiotic prescribing for respiratory illness in young and middle-aged adults.

METHODS

The UK Clinical Practice Research Datalink (CPRD) provided the data source for the study. The CPRD is a database of prospectively collected electronic medical records from approximately 7% of UK general practices. It includes records for all prescriptions issued and medical diagnoses recorded.[9] The study obtained Ethical approval from the South West London Research and Ethics Committee (09/H0806/81) and was approved by the CPRD Independent Scientific Advisory Committee (ISAC protocol 08_083). The study included all CPRD general practices that were included in the cluster trial,[8] as well as sample data for all CPRD general practices that were not included in the trial. All registered patients were included for the trial practices and, in order to provide a manageable dataset for analysis, a random sample of registered patients was taken from non-trial practices. The period of study included the 12 months preceding the start date of the cluster trial withthe date of random allocation was used as the index date.[8] The practices were allocated in five batches between 26th November 2010 and 26th April 2011. For non-trial practices, the median of the allocation dates, 20th January 2011, was used as the index date.

Individual participants were adults aged 18 to 59 years. This was consistent with the eligibility criteria for the trial,[7] which aimed to exclude children and older adults who might be at higher risk of complications. For each participant, we analysed their clinical record for 12 months before the trial index date. General practices were analysed as a single group as there were no overall baseline differences between trial and non-trial practices with respect to consultation and antibiotic prescribing rates.[8] The analysis used 232 general practice Read medical codes (recorded by GPs for each patient who consulted with a RTI), including those for 'colds' and 'upper respiratory tract infection' (URTI); 'cough' and 'bronchitis'; 'sore

For peer review only - http://bmjopen.bmj.com/site/about/guidelines.xhtml

throat', including pharyngitis, laryngitis, tracheitis, epiglottitis and tonsillitis; 'otitis-media' including acute otitis media and otitis media; and 'rhino-sinusitis' including all forms of sinusitis. These were used to identify consultations for acute respiratory tract infections. The source of information for RTI consultations was represented by clinical, referral, and test files data. Only first consultations within an episode were included using a 10 day time window. Therapy file data were used to ascertain antibiotic prescribing information. Antibiotic prescriptions were identified using drug codes that map to section 5.1 of the British National Formulary, excluding drugs used to tuberculosis and leprosy. For each general practice, we estimated the consultation rate for RTI per 1,000 registered patients, the antibiotic prescribing rate per 1,000 registered patients, and the proportion of RTI consultations with antibiotics prescribed as reported previously.[6]

RESULTS

The selection of general practices and patients into the analysis is outlined in Figure 1. There were 582 CPRD general practices available for analysis, 14 practices which contributed fewer than 10 RTI consultations during the study period were excluded leaving 568 for further analysis, including 101 that participated in the trial and 467 that did not participate in the trial. There were 431 practices in England, 21 in Northern Ireland, 66 in Scotland and 50 in Wales. Data were analysed for registered patients aged 18 to 59 years. There were 1,016,779 registered patients with 219,162 consultations for RTI and 118,583 antibiotic prescriptions available for analysis. There was a mean rate of 217 RTI consultations per 1,000 person years and a mean rate of 119 antibiotic prescriptions for RTI per 1,000 person years. Coefficients of variation of the practice-specific rates were 0.30 for the RTI consultation rate and 0.41 for the antibiotic prescribing rate respectively.

For peer review only - http://bmjopen.bmj.com/site/about/guidelines.xhtml

Figure 1 shows the distribution of the practice-specific proportion of RTI consultations with antibiotics prescribed for 568 UK general practices. Considering all RTI consultations as a single group, most practices prescribed antibiotics at between 30% and 80% of RTI consultations. There were only 18 (3%) of practices that prescribed antibiotics at fewer than 30% of RTI consultations and four (1%) of practices that prescribed antibiotics at fewer than 20% of RTI consultations.

Table 1 shows the distribution of the practice-specific prescribing proportions according to the type of RTI consultation. The figures represent the percent of RTI consultations with antibiotics prescribed for the general practice that occupies the stated position in the distribution of results for all 568 practices The median practice prescribed antibiotics at 54% of RTI consultations. The highest prescribing 10% of practices issued prescriptions at 69% or more of RTI consultations, and the highest prescribing 5% of practices issued prescriptions at 74% or more of all RTI consultations. By contrast, the lowest prescribing 10% of practices issued prescriptions at 39% of RTI consultations, and the lowest prescribing 5% of practices issued antibiotic prescriptions at 33% of RTI consultations.

Consultations for ‘cough and bronchitits’ accounted for 39% of RTI consultations; ‘sore throat’, 27%; ‘colds and URTI’, 19%; ‘rhino-sinusitis’ 9%; and ‘otitis media’ 6%. Table 1 shows the distribution of practice specific prescribing proportions according to the type of RTI consultation. The median practice issued antibiotic prescriptions at 38% of consultations for ‘colds’, 48% for ‘cough’, 60% for ‘otitis media’ and ‘sore throat’, and 91% for ‘rhino-sinusitis’. However, the highest prescribing 10% of practices issued antibiotic prescriptions at 72% of consultations for ‘colds and URTI’, 67% for ‘cough and bronchitis’, 78% for ‘sore throat’, 90% for ‘otitis-media’ and 100% for ‘rhino-sinusitis’. The lowest prescribing 10% of

For peer review only - http://bmjopen.bmj.com/site/about/guidelines.xhtml

practices issued antibiotic prescriptions at 14% of consultations for ‘colds and URTI’, 28% for ‘cough’ and 41% for ‘sore throat’.

DISCUSSION

National guidance in the UK recommends that most patients presenting with acute respiratory tract infections can be managed with either no antibiotic prescribing or delayed antibiotic prescribing, with a prescription only being used if symptoms do not improve.[4] The present results show that most general practices in the UK depart substantially from recommended standards of good practice with respect to antibiotic prescribing in a generally low risk age range of young and middle-aged adults. Even for common colds and upper respiratory tract infections, which are generally acknowledged to have a viral aetiology, antibiotics may be prescribed for a third of patients overall and for more than 80% of patients at some general practices. A number of trials have now shown that antibiotic prescribing may be reduced through educational interventions, together with feedback of prescribing information.[10-12] However, these interventions generally have modest effects with generally less than 10-15% reduction in antibiotic prescribing. As Linder[13] has observed, current antibiotic prescribing appears, to be ‘way off the mark’ when viewed in the context of systematic review evidence of lack of benefit[14] and current recommendations for good clinical practice.[4]

For peer review only

Our study had the strengths of a large, representative sample of UK general practices. We acknowledge that we did not include information concerning severity of illness or the presence of comorbidity, which might have accounted for the prescription of antibiotics in some cases. We only analysed prescriptions issued by the practice and it was not possible to estimate from electronic health records whether the prescription was dispensed, or whether a delayed prescribing strategy was intended. There is a Read code for deferred

For peer review only - http://bmjopen.bmj.com/site/about/guidelines.xhtml

antibiotic therapy (8BP0.00) but this was recorded for fewer than 0.5% of medical events. It is unlikely that delayed prescribing can fully account for the high prescribing rates. In an observational study in 13,000 adults with sore throat immediate antibiotics were issued in 42% and 12% given delayed antibiotics.[15] Delayed prescribing is unlikely to vitiate our conclusion that most UK practices prescribe antibiotics to excess. One driver of prescribing is worries about complications but complications are hard to predict and rare and that delayed prescribing is probably as effective as immediate prescribing to reduce the risk of complications.[12] The categories used for analysis may have combined several different entities, for example, prescribing may be more frequent for cases coded as 'bronchitis' than for 'cough'. Prescribing for sinusitis was generally high, even at lower prescribing practices. We have not analysed practice characteristics as possible predictors of antibiotic prescribing, but such analyses typically only explain a small proportion of the variation between practices.[16] The results suggest that most practices commonly prescribe antibiotics unnecessarily. Patient characteristics such as age,[17] gender, comorbidity, smoking status or deprivation category might also be associated with prescribing decisions. Nevertheless, these results suggest that many patients may be prescribed antibiotics unnecessarily. Reducing antibiotic prescribing may lead to lower consultation rates for RTI.[18] The present study did not include children who represent some of the highest users of antibiotic prescriptions[17] but children will be included in a planned cluster randomised trial in CPRD to start in 2015.

The present results have implications for communications with the public as well as for practice prescribing policies. Respiratory infections in this age group are both self-limiting and carry a low risk of complications, moreover the impact of antibiotics on symptom severity and duration is at best marginal. Respiratory infections may be better managed through patient self-care rather than primary care consultation. The high rates of antibiotic prescribing reported by this study indicate a need to shift the entire distribution for antibiotic

For peer review only - http://bmjopen.bmj.com/site/about/guidelines.xhtml

prescribing to lower levels, since there are very few practices that are not prescribing antibiotics to excess, fuelling the development of antibiotic resistance. In addition, there are immediate direct costs from prescribing antibiotics, as well as risks of drug side effects and the perpetuation of unnecessary consultation patterns. There needs to be an active professional debate concerning an overall level of antibiotic utilisation for RTI that might be acceptable, and the size of reduction that individual practices should aim to achieve as a matter of urgency.

For peer review only

Funding:

The study was supported by the Joint Initiative in Electronic Patient Records and Databases in Research, a partnership between the Wellcome Trust, Medical Research Council, Economics & Social Research Council and Engineering & Physical Sciences Research Council (WT086129MA). MG and AD were supported by the National Institute for Health Research (NIHR) Biomedical Research Centre at Guy's and St Thomas' NHS Foundation Trust and King's College London. The views expressed are those of the author(s) and not necessarily those of the NHS, the NIHR or the Department of Health. CPRD has received funding from the MHRA, Wellcome Trust, Medical Research Council, NIHR Health Technology Assessment programme, Innovative Medicine Initiative, UK Department of Health, Technology Strategy Board, Seventh Framework Programme EU, various universities, contract research organisations and pharmaceutical companies. The views expressed in this paper are those of the authors and do not reflect the official policy or position of the MHRA.

Competing interests:

The authors declare that they have no competing interests.

For peer review only - http://bmjopen.bmj.com/site/about/guidelines.xhtml

Contributor statement:

MG designed, supervised, and drafted the paper. LM assisted with draft and conclusions. JC and AD contributed to data analysis. MA, MM, PL, LY contributed to design, write-up and interpretation of data. TvS and GM contributed to practice recruitment and facilitated access to CPRD. All authors contributed to the paper and approved the final draft. All authors read and approved the final manuscript.

Data sharing:

There is no data sharing available at present.

For peer review only

For peer review only - http://bmjopen.bmj.com/site/about/guidelines.xhtml

REFERENCES

1. Chief Medical Officer. *Annual Report of the Chief Medical Officer. Volume 2, 2011. Infections and the rise of antimicrobial resistance.* London: Department of Health, 2013.

2. Little P, Gould C, Williamson I et al. Reattendance and complications in a randomised trial of prescribing strategies for sore throat: the medicalising effect of prescribing antibiotics. *BMJ*. 1997;**315**:350-352.

3. Glasziou PP, Del Mar CB, Sanders SL et al.. Antibiotics for acute otitis media in children. *The Cochrane database of systematic reviews*. 2004 (1):CD000219.

4. National Institute for Health and Care Excellence. *Prescribing of antibiotics for self-limiting respiratory tract infections in adults and children in primary care. Final scope 130907*. London: National Institute for Health and Care Excellence; 2008.

5. Ashworth M, Latinovic R, Charlton J et al. Why has antibiotic prescribing for respiratory illness declined in primary care? A longitudinal study using the General Practice Research Database. *J Public Health*. 2004;**26**:268-274.

6. Gulliford M, Latinovic R, Charlton J et al. Selective decrease in consultations and antibiotic prescribing for acute respiratory tract infections in UK primary care up to 2006. *J Public Health*. 2009;**31**:512-520.

7. Gulliford MC, van Staa T, McDermott L et al. Cluster randomised trial in the General Practice Research Database: 1. Electronic decision support to reduce antibiotic prescribing in primary care (eCRT study). *Trials*. 2011;12:115.

8. Gulliford M, van Staa T, Dregan A et al. Utilising electronic health records for intervention research. Cluster randomised trial to reduce antibiotic prescribing in primary care (eCRT study). *Annals of Family Medicine*. 2014;**12**: 344-51.

For peer review only - http://bmjopen.bmj.com/site/about/guidelines.xhtml

9. Williams T, van Staa T, Puri S et al.. Recent advances in the utility and use of the General Practice Research Database as an example of a UK Primary Care Data resource. *Therapeutic Advances in Drug Safety*. 2012;**3:**89-99.

10. Little P, Stuart B, Francis N et al. Effects of internet-based training on antibiotic prescribing rates for acute respiratory-tract infections: a multinational, cluster, randomised, factorial, controlled trial. *Lancet*. 2013;**382**:1175-82.

11. Butler CC, Simpson SA, Dunstan F et al. Effectiveness of multifaceted educational programme to reduce antibiotic dispensing in primary care: practice based randomised controlled trial. *BMJ*. 2012;**344**:d8173.

12. Gonzales R, Anderer T, McCuloch CE et al. A cluster randomized trial of decision support strategies for reducing antibiotic use in acute bronchitis. *JAMA Internal Medicine*. 2013;**173**:267-273.

13. Linder JA. Antibiotic Prescribing for acute respiratory infections--success that's way off the mark: comment on "A cluster randomized trial of decision support strategies for reducing antibiotic use in acute bronchitis". *JAMA Intern Med*. 2013;**173**:273-5.

14. Del Mar CB, Glasziou PP and Spinks AB. Antibiotics for sore throat. *Cochrane Database Syst Rev*. 2000:CD000023.

15. Little P, Stuart B, Hobbs FD et al. Antibiotic prescription strategies for acute sore throat: a prospective observational cohort study. *Lancet Infect Dis*. 2014;**14**:213-9.

16. Boggon R, Hubbard R, Smeeth L et al. Variability of antibiotic prescribing in patients with chronic obstructive pulmonary disease exacerbations: a cohort study. *BMC Pulmonary Medicine*. 2013;**13**:32.

17. Ashworth M, Charlton J, Latinovic R et al. Age-related changes in consultations and antibiotic prescribing for acute respiratory infections, 1995–2000. Data from the UK General

For peer review only - http://bmjopen.bmj.com/site/about/guidelines.xhtml

Practice Research Database. *Journal of Clinical Pharmacy and Therapeutics*. 2006;**31**:461-467.

18. Ashworth M, Charlton J, Ballard K et al. Variations in antibiotic prescribing and consultation rates for acute respiratory infection in UK general practices 1995-2000. *BrJ GenPract*. 2005;**55**:603-608.

For peer review only

For peer review only - http://bmjopen.bmj.com/site/about/guidelines.xhtml

Table 1: Centiles of the distribution of the proportion (%) of RTI consultations with antibiotics prescribed at 568 UK general practices.

	Proportion of RTI consultations with antibiotics prescribed at UK general practices						
	Lowest 5% of practices	Lowest 10%	Lowest 25%	Median	Highest 25%	Highest 10%	Highest 5% of practices
All	33	39	46	54	63	69	74
'Colds' and 'URTI'	9	14	25	38	56	72	81
'Cough and bronchitis'	22	28	38	48	59	67	71
'Otitis-media'	22	32	45	60	75	90	100
'Rhino-sinusitis'	67	75	83	91	98	100	100
'Sore throat'	35	41	50	60	68	78	83

URTI, upper respiratory tract infection
Note that one practice may not occupy the same centile of prescribing for each condition.

For peer review only - http://bmjopen.bmj.com/site/about/guidelines.xhtml

For peer review only

For peer review only - http://bmjopen.bmj.com/site/about/guidelines.xhtml

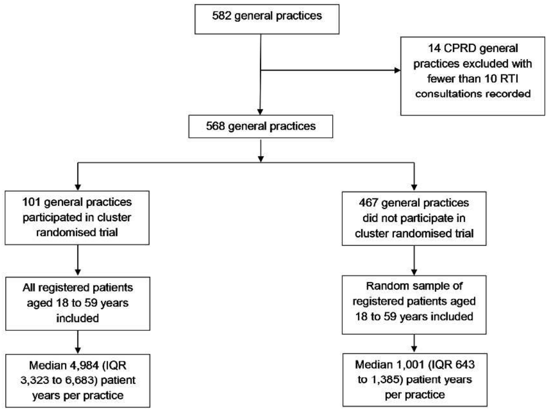

Figure 1. Flowchart showing selection of general practices and participants. (IQR, interquartile range).
338x190mm (300 x 300 DPI)

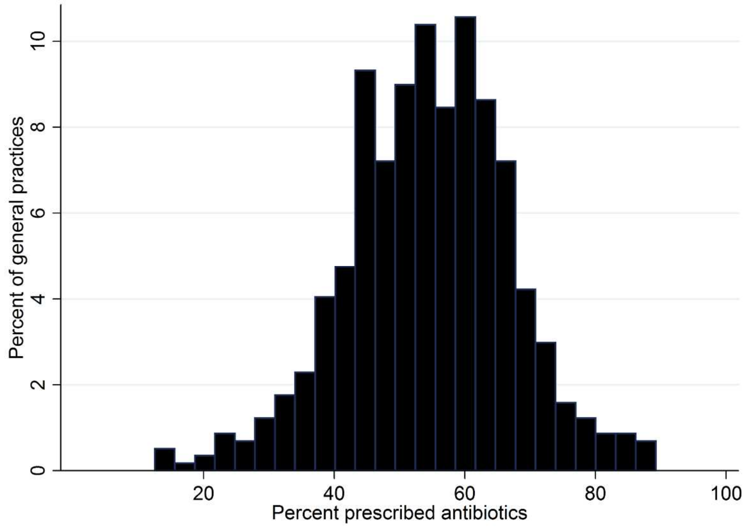

Figure 2: Distribution for percent of RTI consultations with antibiotics prescribed for adults aged 18 to 59 years at 568 UK general practices.

338x190mm (300 x 300 DPI)

For peer review only
